# Management of post-analytical processes in the clinical laboratory according to ISO 15189:2012. Considerations about the management of clinical samples, ensuring quality of post-analytical processes, and laboratory information management

**DOI:** 10.1515/almed-2021-0044

**Published:** 2021-05-31

**Authors:** Mᵃ Libòria López Yeste, Antonia R. Pons Mas, Leonor Guiñón Muñoz, Silvia Izquierdo Álvarez, Fernando Marqués García, Aurora Blanco Font, Natalia F. Pascual Gómez, Lorena Sánchez Gancedo, Ana García Álvarez, Francisco A. Bernabeu Andreu, Mᵃ Patrocinio Chueca Rodríguez, Luisa Álvarez Domínguez

**Affiliations:** CATLAB, Viladecavalls, Barcelona, Spain; Clinical Analysis Service, Hospital Universitari Son Espases, Mallorca, Spain; Quality Department, Laboratories, Hospital de la Santa Creu i Sant Pau, Barcelona, Spain; Service of Clinical Biochemistry, Hospital Universitario Miguel Servet, Zaragoza, Spain; Clinical Biochemistry Department, Metropolitan North Clinical Laboratory (LUMN), Germans Trias i Pujol Universitary Hospital, Badalona, Barcelona, Spain; Laboratori Clínic Territorial Metropolitana Sud, Hospital Universitari de Bellvitge, Barcelona, Spain; Clinical Analysis Service, Hospital Universitario de la Princesa, Madrid, Spain; Quality, Institute of Oncologic and Molecular Oncology, Asturias, Spain; Clinical Analysis Service, Hospital Clínico San Carlos, Madrid, Spain; Service of Clinical Analysis – Clinical Biochemistry, Hospital Universitario Puerta de Hierro, Majadahonda, Madrid, Spain; Laboratory Accreditation Board of the Spanish Society of Laboratory Medicine, Tudela (Navarra), Spain; Laboratory Accreditation Board of the Spanish Society of Laboratory Medicine, Barcelona, Spain

**Keywords:** accreditation, clinical laboratory, ISO 15189 standard, laboratory information system, post-analytical

## Abstract

ISO 15189:2012 establishes the requirements for clinical sample management, ensuring quality of process and laboratory information management. The accreditation authority, ENAC in Spain, established the requirements for the authorized use of the label in reports issued by accredited laboratories. These recommendations are applicable to the postanalytical processes and the professionals involved. The Standard requires laboratories to define and document the duration and conditions of sample retention. Laboratories are also required to design an internal quality control scheme to verify whether postanalytical activities attain the expected standards. Information management requirements are also established and laboratories are required to design a contingency plan to ensure the communication of laboratory results. Instructions are finally provided about the correct use of the accreditation label in laboratory reports. A range of nations and scientific societies support that clinical laboratories should be required to obtain accreditation. With ISO 15189 being the most specific standard for demonstrating technical performance, a clear understanding of its requirements is essential for proper implementation.

## Introduction

Clinical laboratories increasingly devote more efforts to improve their methodological and communication skills to help physicians in the interpretation of test results. Added to UNE-EN ISO 15189:2013 (hereinafter, the ISO 15189 Standard) requirements for the revision, reporting and release of clinical test results, described elsewhere [1], this Standard also establishes other requirements for postanalytical processes. These requirements address sample storage, retention and disposal, the inclusion of postanalytical processes in laboratory quality assurance and continuous improvement, laboratory information management, and the need for a contingency plan that ensures the communication of test results in any scenario [2], [3]. ISO 15189 is based on laboratory best practices and employs the information obtained from the quality management system to generate corrective and improvement actions.

ISO 15189 establishes a set of requirements for laboratories to implement effective methods for the detection and classification of postanalytical errors and the incorporation of information systems and standard operating procedures aimed at reducing errors [4]. Special emphasis is placed on the communication of results, laboratory information management and risk management. The standard also requires that a contingency plan is designed. Additionally, and considering that the use of the ENAC label in laboratory reports is the way laboratories demonstrate that they comply with accreditation requirements, it is important to pay attention on the ENAC document CEA-ENAC-01 “Requirements for the use of the ENAC label and reference of certification”, which establishes the requirements for label use, which must be known to all accredited laboratories (latest version available at www.enac.es).

These recommendations do not extend the Standard and should be considered complementary information that facilitates its interpretation and implementation. The scope of application is the staff involved in postanalytical processes in the clinical laboratory.

## Sample storage, retention and disposal

**Figure 1:**
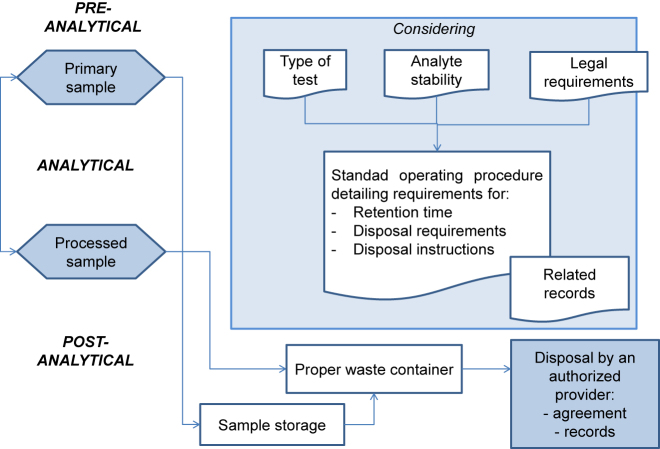
Sample storage, retention and disposal flowchart.

ISO 15189 requires that the laboratory defines and documents the duration of sample retention, as well as sample storage and discard conditions. The standard operating procedure should specify the duration of sample retention and sample storage conditions, which will be defined according to the nature and stability of each analyte [5] and the applicable legal requirements (see Figure 1). The duration of sample storage may be extended by legal requirements associated with some types of studies (i.e. histological analyses, gene testing, pediatric studies).

Access to archive samples must be restricted. Easy sample traceability and location for retrieval must be ensured (for additional testing, result verification, and/or legal requirement, among others). Therefore, it is recommended that the date and person responsible for sample retrieval be recorded, which is essential in case it is required by law.

The procedure for disposal of clinical samples and consumables must be documented by the laboratory. It is laboratory’s responsibility to comply with the laws and regulations in relation to the prevention of occupation hazards, even though waste disposal is performed by an authorized external supplier.

## Quality assurance and continuous improvement

As it occurs with preanalytical and analytical procedures, postanalytical procedures must be evaluated and audited to ensure compliance with standards. Auditing is also intended to verify whether this process satisfies user’s needs and requirements. Thus, efforts are necessary to improve the effectiveness of the postanalytical process.

Evaluation of the postanalytical process must include the activities carried out both, in the laboratory (post-analytical phase) and out of the laboratory (post-postanalytical phase). A range of studies have demonstrated that most errors occur in the post-postanalytical phase due to the variety of services and number of external professionals involved [6].

The incidence of errors in the post-analytical process varies significantly from 18 [7] to 47% [8] of total errors. The most frequent errors include misinterpretation of test results by the laboratory, delayed delivery of reports, loss of reports, and failure to report sample-related events to the requesting physician. Postanalytical errors may lead to incorrect clinical decisions based on a misinterpretation of test results. Poor decision-making may affect the clinical course, prognosis and outcome of the patient. These are traditionally called post-postanalytical phase/process errors [7], [8], [9]. These processes can be improved by enhancing interaction between the professionals involved, where communication and training play a crucial role [10]. Another potential source of errors that is expected to increase in the future is the inability to check test results on the electronic medical history of the patient due to technical issues.

**Figure 2:**
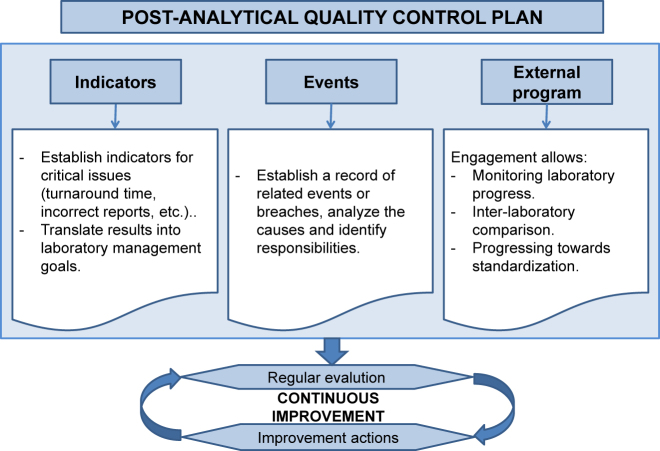
Characteristics of the post-analytical quality control plan.

The laboratory is required to design a quality control plan to verify whether post-analytical activities meet the established standards. This control is primarily based on strategies aimed at detecting errors and establishing quality indicators (see Figure 2).

Quality indicators of postanalytical processes allow for the objective evaluation of the service delivered to ensure the quality of this process based on compliance with quality standards. For a proper evaluation of results, it is essential that communication between laboratory staff and ordering physicians is regular, fluid and based on a standard protocol. Indicators of the postanalytical process include reference intervals, cut-off points, action points, graphic representation, self-validation, interpretative comments, reflex tests (tests automatically performed on the basis of an algorithm) or reflective tests (tests added by a professional considering the clinical context) [11], [12], the clinical information required for the correct interpretation of results, reporting of critical values, and effective release of laboratory test results through the LIS [13]. According to numerous users, the most important indicator of laboratory performance is the time period between a test request and delivery of results (turnaround time), which is especially relevant for patients seen in or admitted to emergency care.

A model of quality indicators was presented at the Consensus Conference held in Padua in 2013, which assigns a priority score to each indicator to help laboratories implement effective interventions to improve performance. A criterion was proposed for the establishment of quality standards to assess laboratory performance [13]. In the 2016 Padua Conference, a review was performed of the results obtained since 2014 to identify the indicators and quality standards that should be established to comply with ISO 15189:2012 requirements through continuous monitoring of critical activities to minimize risks. The established indicators evaluate all postanalytical stages, from result validation to the effective, timely result release. Indicators are expressed as a percentage of errors in each laboratory activity. The way each activity is expressed is essential (number of requests, patients, samples, among others), since it will allow interlaboratory comparison (benchmarking) or comparison against recommendations of scientific societies and entities. The indicators included in the Padua consensus statement are detailed in Table 1.

**Table 1:** Indicators included in the Padua Consensus Statement on the post-analytical process [15].

Activity	Description	Calculation formula
Turnaround time	Number of reports issued not complying with the agreed timeframe with respect to the total number of reports.	Number of out-of-time reports/total number of reports in a year × 100 (%).
Turnaround time	Turnaround time for different analytes (potassium, INR, WBC, troponin I or T) from request receipt to result report.	Day and time of issue – day and time of entry (day, time (h/min)) “… for non-emergency analysis of serum potassium concentrations”.
Corrected laboratory reports	Percentage of reports corrected by the laboratory after delivery with respect to the total number of reports.	Number of corrected reports in a year/total number of reports in a year × 100 (%).
Critical result notification	Critical results reported out of time with respect to the total number of reported results.	Number of critical results reported out of time in a year/Total number of critical results in a year × 100 (%) “… for all tests with indication of emergency report”;… for non-emergency analysis of serum potassium concentrations, etc.”
Critical result notification	Mean time to critical result notification.	Mean (time of result notification – time of result release to the ordering physician (min)” “… for all tests with indication of emergency report”;… for non-emergency analysis of serum potassium concentrations, etc.”

For further information, visit: https://www.ifcc.org/media/455725/Quality_Indicators_Key_Processes.pdf [15].

To overcome these problems and build up a picture of total laboratory errors, the Working Group on “Laboratory Errors and Patient Safety” (WG-LEPS) of the International Federation of Clinical Chemistry and Laboratory Medicine (IFCC) implemented in 2008 a project to design a model of quality indicators (MQI), a harmonized method for data collection administered as an External Quality Assurance Program (EQAP) where confidentiality is guaranteed [14], [15].

The laboratory will also take part in a benchmarking program. Despite the lack of consensus on quality standards, the relevance of joining an EQAP relies on the information these programs provide about compliance with the standards established by each laboratory and allow interlaboratory comparison. Despite the high number of errors that occur throughout the postanalytical process and the fact that it is a requirement of ISO 15189 (5.6.3), EQAPs are not yet fully developed. In the recent years, some entities are implementing EQAPs in parallel to benchmarking programs for preanalytical processes. These programs include the benchmarking program designed by the WG-LEPS of the IFCC, active since 2008, which evaluates the model of indicators designed by the WG-LEPS itself. Although it is infrequent, some benchmarking and RCPA QAP and UK NEQAS programs address the topic of interpretative comments in the field of Clinical Biochemistry [6].

## Laboratory information management

**Figure 3:**
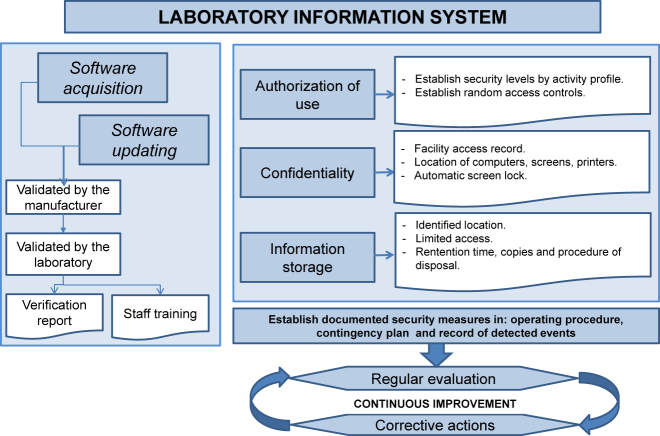
Main laboratory information management requirements.

State-of-the art technologies and the increased amount of data managed at the clinical laboratory have led to the establishment of demanding requirements for laboratory information management systems [16]. With this respect, ISO 15189:2012 requires that all laboratories have an information management system that ensures the accessibility, integrity, security and confidentiality of patient data. It is essential that both, computer-based and paper-based information systems are considered, as well as all scenarios where information is received, generated or reported. Figure 3 summarizes the main ISO 15189:2012 requirements.

The laboratory must guarantee patient data protection by adopting all measures necessary to prevent any unauthorized access to data (i.e. access to facilities, location of computers, automatic screen lock, to name a few). With regard to laboratory reports, all methods of delivery must be considered (electronic systems, paper, electronic mail or smartphone) to establish the appropriate security measures (dissociated information, encryption, among others).

The laboratory should identify the persons who are authorized to access and use laboratory information and establish security levels based on user’s profile.

Security measures must be adopted to protect information and prevent any alteration or destruction of information by unauthorized personnel. Monitoring of electronic systems allows for the detection of potential breaches of security.

Measures have to be adopted to mitigate the risk of unintended information degradation or loss, either if it is available on paper or electronically.

The information system must have been validated by the supplier. Yet, the laboratory must verify and monitor the correct transfer of unaltered information between the laboratory and other internal (i.e. laboratory analyzers) and external (i.e. HIS) information systems. In some situations, verification is also required, such as after software updating or after a piece of equipment has long been unused. This validation/verification should be documented in a report.

Availability and access to all the information generated must also be ensured. The location of stored information must also be clearly identified, and access must be restricted to authorized personnel, who will be able to recover information at any time it is necessary.

Information must be stored in a secure system with enough storage space (i.e. for analyzer information backup). Time of document retention must be defined and documented by the laboratory in compliance with legal requirements. The procedure for document identification and disposal must also be established in accordance with the established retention time while confidentiality is guaranteed. This procedure can be either performed by the laboratory or by an authorized supplier upon previous agreement.

## Contingency result communication plan

Section 5.10.3 of the ISO 15189 standard on information system management establishes that the laboratory must design and document contingency plans to guarantee the provision of its services in case of technical fault or interruption.

If there is a HIS or LIS failure, the ordering physician will activate the contingency plan and inform the heads of the involved hospital services.

The contingency plan must consider different scenarios and clearly define the actions to be taken and the professional responsible for each action [17]. Situations that may arise include a failure of the HIS or/and LIS or the interruption of communication between the HIS and LIS. These situations may be solved by using paper test requests, manually entering data into the LIS, scheduling tests in the different analyzers, printing results from the different analyzers and their interpretative comments, sending to the requesting Service a copy of the test request with the results attached, or by remote printing of reports in printers for that intended use. Once the event has been solved, the results obtained from the different analyzers must be entered into the LIS and the laboratory must verify that all requests are complete.

Other potential scenarios include unauthorized access to the hospital or laboratory information system (hackers), a failure of the hospital telephone line, which will involve the use of emergency phones in the laboratory and emergency care services connected to the power grid, or a failure in the pneumatic tube, which will involve sending samples through porters.

Other situations in which laboratory services may be interrupted include the breakdown of an analyzer system, auxiliary equipment, refrigerator, or freezer; interruption of the electricity or water supply; or other exceptional situations (for example, meteorological). In these situations, the laboratory must guarantee the continuity of its services by adding annexes to the general plan or designing different contingency plans for some specific areas; these plans must clearly establish priorities and work coordination tasks and evaluate the human resources needed. These plans will also specify the procedure for manual sample entry, storage for later processing, testing in duplicate analyzers or systems, or shipping samples to other facilities under proper conditions, where applicable.

**Figure 4:**
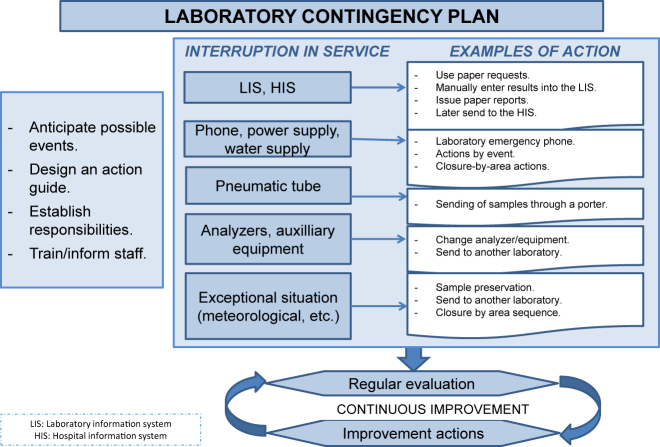
Characteristics of the clinical laboratory contingency plan.

The characteristics of the contingency plan are detailed in Figure 4.

## Use of the ENAC label in result reports

Accreditation entities, as ENAC in Spain, establish the conditions for accredited laboratories to give proof of their accreditation by the use of the ENAC label.

As a proof of accreditation, reports of results obtained in an accredited activity will be marked with the ENAC label. As described in the CEA-ENAC-01 [18] document, the ENAC label is the combination of the ENAC trademark (or the accreditation reference), the accredited activity, and the accreditation number.

The label is always associated with the name or trademark of the accredited organization and should appear at least on the first page of the report. If accredited activities are performed in different facilities of the organization, the facility where the activity was performed should be specified.

When the result report includes an accredited activity, the ENAC label can be used or a statement can be included in the report such as “ENAC-accredited Laboratory with reference number…”, as established in Section 11.2 of document CEA-ENAC-01. The statement must be written with the same font size and style as the ones used in the body of the report and be readable.

To avoid confusion and make accredited results easily identifiable, when not all activities are accredited:

– Mark with a symbol (asterisk or similar) the analytes that are not accredited and include a legend in a visible place near the label indicating that the marked analytes are not accredited.
– Alternatively, place a symbol or a legend next to the accredited analytes indicating its accreditation status. In case a symbol is used, add a legend in the report.

In some circumstances, a report may not bear the ENAC label although it contains results for accredited analytes. In such event, prior to the delivery of results, customers will be informed of such circumstance and its associated consequences and give consent. These cases are exceptional, as when there is prior explicit request and approval of the ordering physician (in this case, the accredited organization will inform the customer that a report not bearing the ENAC label is not considered accredited to all effects); when, for technical reasons, the accredited organization cannot temporarily comply with one or several accreditation requirements (in these cases, an explanatory note will be included in the report), or when placing the label in the result report is not possible (i.e. when the customer displays results on a web portal).

When an accredited laboratory reports results of activities performed in an external laboratory, these activities will be clearly identified and labeled as ENAC-accredited or non-accredited activities.

For further information about the use of the ENAC label, see the CEA-ENAC-01 document.

Table 2 summarizes the main indications for the use of the label in result reports.

**Table 2:** Main indications for the use of the ENAC label in result reports.

– If the report includes an accredited activity: include the label or explanatory note detailing the accreditation number.
– When only some of the analytes reported are accredited, identify either: – Not accredited analytes or – Accredited analytes. Include an explanatory note next to the analyte or on a footnote providing the accreditation number. – The label cannot be used if none of the reported activities are accredited.
– Issue of unlabelled reports: – On express customer’s request. – When the laboratory is temporarily unable to comply with accreditation requirements. Include an explanatory note in the report. – When use is not feasible (visualization on a web portal).
– Activities performed by external suppliers: – Accredited activity performed by an external accredited laboratory: clearly identify the activities performed by an external laboratory. – Non-accredited activity performed by an accredited external laboratory: an explanatory note may be included indicating the accrediation number of the external laboratory, but the label cannot be used.
– Flexible scope: accredited tests are shown in the list of accredited tests classified by categories.
